# Association of Pace of Aging Measured by Blood-Based DNA Methylation With Age-Related Cognitive Impairment and Dementia

**DOI:** 10.1212/WNL.0000000000200898

**Published:** 2022-09-27

**Authors:** Karen Sugden, Avshalom Caspi, Maxwell L. Elliott, Kyle J. Bourassa, Kartik Chamarti, David L. Corcoran, Ahmad R. Hariri, Renate M. Houts, Meeraj Kothari, Stephen Kritchevsky, George A. Kuchel, Jonathan S. Mill, Benjamin S. Williams, Daniel W. Belsky, Terrie E. Moffitt, for the Alzheimer's Disease Neuroimaging Initiative*

**Affiliations:** From the Department of Psychology and Neuroscience (K.S., A.C., M.L.E., K.C., A.R.H., R.M.H., B.S.W., T.E.M.), and Center for Genomic and Computational Biology (K.S., A.C., B.S.W., T.E.M.), Duke University, Durham, NC; Department of Psychiatry and Behavioral Sciences (A.C., T.E.M.), Duke University School of Medicine, Durham, NC; Social, Genetic, and Developmental Psychiatry Centre (A.C, T.E.M.), Institute of Psychiatry, Psychology, and Neuroscience, King's College London, UK. Center for the Study of Aging and Human Development (K.J.B.), Duke University, Durham, NC; Department of Genetics (D.L.C.), University of North Carolina School of Medicine, Chapel Hill; Butler Columbia Aging Center (M.K., D.W.B.), Columbia University, New York, New York; Sticht Center for Healthy Aging and Alzheimer's Prevention (S.K.), Wake Forest School of Medicine, Winston-Salem, NC; UConn Center on Aging (G.A.K.), University of Connecticut, Farmington, Connecticut, USA; College of Medicine and Health (J.S.M.), University of Exeter Medical School, Devon, UK; and Department of Epidemiology (D.W.B.), Columbia University Mailman School of Public Health, New York, New York.

## Abstract

**Background and Objectives:**

DNA methylation algorithms are increasingly used to estimate biological aging; however, how these proposed measures of whole-organism biological aging relate to aging in the brain is not known. We used data from the Alzheimer's Disease Neuroimaging Initiative (ADNI) and the Framingham Heart Study (FHS) Offspring Cohort to test the association between blood-based DNA methylation measures of biological aging and cognitive impairment and dementia in older adults.

**Methods:**

We tested 3 “generations” of DNA methylation age algorithms (first generation: Horvath and Hannum clocks; second generation: PhenoAge and GrimAge; and third generation: DunedinPACE, Dunedin Pace of Aging Calculated from the Epigenome) against the following measures of cognitive impairment in ADNI: clinical diagnosis of dementia and mild cognitive impairment, scores on Alzheimer disease (AD) / Alzheimer disease and related dementias (ADRD) screening tests (Alzheimer's Disease Assessment Scale, Mini-Mental State Examination, and Montreal Cognitive Assessment), and scores on cognitive tests (Rey Auditory Verbal Learning Test, Logical Memory test, and Trail Making Test). In an independent replication in the FHS Offspring Cohort, we further tested the longitudinal association between the DNA methylation algorithms and the risk of developing dementia.

**Results:**

In ADNI (*N* = 649 individuals), the first-generation (Horvath and Hannum DNA methylation age clocks) and the second-generation (PhenoAge and GrimAge) DNA methylation measures of aging were not consistently associated with measures of cognitive impairment in older adults. By contrast, a third-generation measure of biological aging, DunedinPACE, was associated with clinical diagnosis of Alzheimer disease (beta [95% CI] = 0.28 [0.08–0.47]), poorer scores on Alzheimer disease/ADRD screening tests (beta [Robust SE] = −0.10 [0.04] to 0.08[0.04]), and cognitive tests (beta [Robust SE] = −0.12 [0.04] to 0.10 [0.03]). The association between faster pace of aging, as measured by DunedinPACE, and risk of developing dementia was confirmed in a longitudinal analysis of the FHS Offspring Cohort (*N* = 2,264 individuals, hazard ratio [95% CI] = 1.27 [1.07–1.49]).

**Discussion:**

Third-generation blood-based DNA methylation measures of aging could prove valuable for measuring differences between individuals in the rate at which they age and in their risk for cognitive decline, and for evaluating interventions to slow aging.

Aging can be conceptualized as gradual and progressive deterioration in biological system integrity causing morbidity and disability.^1^ These changes, in turn, are believed to increase vulnerability to multiple age-related diseases,^2,3^ including dementias. Advances in both basic and applied aging research could be spurred by the availability of tools that can measure biological aging. Medical, behavioral, and social sciences need measures of biological aging to identify risk factors and mechanisms that accelerate aging and to use in studies of social groups that are believed to be aging at different rates.^4^ Applied science needs measures of biological aging to evaluate whether interventions succeed in slowing aging. Multiple companies are developing drug therapies that target aging biology, and several are being evaluated in human trials (clinicaltrials.gov). Behavior-change science is also working toward interventions to extend healthspan, including increasing physical activity, hypertension control, cognitive stimulation, dietary modification, and social engagement.^5-7^ Whether they are pharmaceutical or behavioral, interventions that aim to extend healthspan need to have a measure to evaluate whether aging has indeed been slowed. However, as of this writing, there is no widely accepted measure of biological aging.^8,9^ This article reports the association between dementia, one of the most feared and costly diseases of aging, and 5 leading candidate measures of aging based on DNA methylation.

DNA methylation is an epigenetic mechanism by which specific points of the genome (CpGs) are chemically modified (methylated) and thereby affect gene regulation. Recent efforts to develop measures of aging have focused on blood DNA methylation because it is a biological substrate that is sensitive to age-related changes.^10,11^ Using machine learning, these measurement efforts involve developing algorithms to capture information about aging by using data about DNA methylation levels of multiple CpGs across the genome. These methylation algorithms have evolved rapidly. The first generation of methylation algorithms was trained on chronologic age in samples ranging from children to older adults. These “clocks” identified methylation patterns that vary by chronologic age. If a person's score on such clocks is older than his/her actual age, it is inferred that s/he is biologically older. The first-generation algorithms include the “Hannum clock”^12^ and the “Horvath clock.”^13^ The second generation of methylation algorithms added measures of people's current physiologic status to identify methylation patterns that account for differences in current health and that predict mortality. These second-generation algorithms include PhenoAge^14^ and GrimAge.^15^ The DunedinPACE (Pace of Aging Calculated from the Epigenome) is a third-generation algorithm that was developed by first measuring people's rate of physiologic change over time and then identifying the methylation patterns that captured individual differences in their age-related decline. Specifically, it measured age-related change in 19 biomarkers among individuals of the same chronologic age over a 20-year observation period^16^ and then identified methylation patterns at the end of the observation period that estimated how fast aging occurred during the years leading up to the point of measurement.^17^ Thus, it is designed to capture methylation patterns reflecting individual differences in age-related decline.

These DNA methylation algorithms have been embraced by the research community and private companies. However, the literature evaluating them is fragmented. Although all of these algorithms purport to measure aging, they have surprisingly low agreement^18,19^; articles often report promising findings from one (or more) DNA methylation algorithms, but often in different samples, and many algorithms show inconsistent associations with outcomes.^11,20-23^ Important validation steps are now being taken to rigorously evaluate multiple DNA methylation algorithms in the same study (e.g., in the Health and Retirement Survey).^19,24^ What has not been reported is an evaluation of multiple DNA methylation algorithms in the same study with the outcome of dementia.

Here, we leverage data from the Alzheimer's Disease Neuroimaging Initiative (ADNI) to test associations of the leading measures from the 3 generations of DNA methylation algorithms with cognitive aging and dementia. We examined 3 sets of “gold standard” measurements in cognitive-aging research. First, we compared the DNA methylation algorithms' scores as a function of ADNI participants' diagnoses: cognitively normal (CN), mild cognitive impairment (MCI), or Dementia. Second, we evaluated the algorithms in relation to 3 instruments that are used as cognitive screening tools for Alzheimer disease (AD) / Alzheimer disease and related dementias (ADRD): the Alzheimer's Disease Assessment Scale–Cognitive (ADAS-Cog-13^25^), the Mini-Mental State Examination (MMSE^26^), and the Montreal Cognitive Assessment (MoCA^27^). Third, we evaluated the algorithms in relation to well-established tests of learning and memory (Rey Auditory Verbal Learning Test^28^), episodic memory (Logical Memory test^29^), and executive function (Trail Making Test^30^) that are known to decline with age. We then turned to a second independent sample, the Framingham Heart Study (FHS) Offspring Cohort, to evaluate whether and which DNA methylation measures of biological aging could longitudinally predict risk of developing dementia.

## Methods

### The ADNI DNA Methylation Sample

Data were obtained from the ADNI database. The primary goal of ADNI has been to test whether MRI, PET, other biological markers, and clinical and neuropsychological assessment can be combined to measure the progression of MCI and early AD. Inclusion and exclusion criteria included Hachinski Ischemic Score^31^ ≤4, Geriatric Depression Scale score^32^ <6, visual and auditory acuity adequate for neuropsychological testing, good general health with no diseases precluding enrollment, sixth grade education or work-history equivalent, no medical contraindications to MRI, no psychoactive medications that affect cognitive function, medications stable for 4 weeks before screening, and not enrolled in other trials or studies concurrently.^33^ Data were downloaded from the ADNI data repository (adni.loni.usc.edu) on June 3, 2020.

### DNA Methylation Data

DNA methylation was measured in DNA samples from whole blood using Illumina 450k arrays and run at the University of Minnesota and Johns Hopkins University (dbGaP phs000724.v9.p13).

DNA methylation was measured in DNA from whole blood using the Illumina Infinium HumanMethylationEPIC BeadChip Array and run at AbbVie. A total of 649 ADNI participants had methylation data. Participants varied on the number of repeat DNA methylation measurements they had: 83 had only a baseline measurement; 121 had 2 measurements (baseline plus 1 repeat), mean 14.5 months between measurements (SD = 7.06); 407 had 3 measurements, mean 12.1 months between measurements (SD = 1.5); 29 had 4, mean 10.4 months between measurements (SD = 3.54); and 9 had 5, mean 12.5 months between measurements (SD = 2.25). Samples were randomized using a modified incomplete balanced block design, whereby all samples from a participant were placed on the same chip, with remaining chip space occupied by age-matched and sex-matched samples. Participants from different diagnosis groups were placed on the same chip to avoid confounding.

DNA methylation data were subjected to QC by ADNI investigators before receipt. Samples with missing rate >1% at *p* < 0.05, poor single nucleotide polymorphism (SNP) matching to the 65 SNP control probe locations, and uncertain sex were excluded. Filtered data were normalized using the “NormalizeMethylumiSet” function in the *R* package *Methylumi*. Before normalization, replicate samples (test-retest of the same sample, *N* = 198) were identified and removed from the data set. Probes were removed if detection the *p*-value was >0.05 in more than 10% of individuals (*N* = 611 probes).

A flowchart documenting the number of samples at each stage of data preparation is shown in eFigure 1A (links.lww.com/WNL/C174).

### Cognitive Assessments

Data about diagnosis, cognitive impairment screening tests, and cognitive function were extracted from data tables available in the “*ADNIMERGE*” package in *R* and then cross-matched to participants with available DNA methylation data. Measures are described below.

#### Diagnosis

Diagnosis was made by a study physician at the time of assessment and categorized as CN, MCI, and AD-dementia.

#### Clinical Assessment

The ADAS-Cog-13 is a structured scale that evaluates memory, reasoning, language, orientation, ideational praxis, and constructional praxis.^25^ Delayed Word Recall and Number Cancellation are included in addition to the 11 standard ADAS-Cog Items.^34^ The test is scored for errors, ranging from 0 (best performance) to 85 (worse performance). The MMSE is a screening instrument that evaluates orientation, memory, attention, concentration, naming, repetition, comprehension, and ability to create a sentence and to copy 2 overlapping pentagons.^26^ The MMSE is scored as the number of correctly completed items ranging from 0 (worse performance) to 30 (best performance). The MoCA is designed to detect individuals at the MCI stage of cognitive dysfunction.^27^ The scale ranges from 0 (worse performance) to 30 (best performance).

#### Cognitive Function

The Rey Auditory Verbal Learning Test is a list learning task which assesses learning and memory. On each of 5 learning trials, 15 unrelated nouns are presented orally at the rate of 1 word per second and immediate free recall of the words is elicited. After a 30-minute delay filled with unrelated testing, free recall of the original 15-word list is elicited. Both immediate recall and the percent forgotten are used. The Logical Memory tests I and II (Delayed Paragraph Recall) is from the Wechsler^29^ Memory Scale–Revised. Free recall of 1 short story is elicited immediately after being read aloud to the participant and again after a 30-minute delay. The total bits of information recalled after the delay interval (maximum score = 25) are analyzed. The Trail Making Test, Part B, consists of 25 circles, either numbered (1 through 13) or containing letters (A through L). Participants connect the circles while alternating between numbers and letters (e.g., A to 1; 1 to B; B to 2; 2 to C). Time to complete (300 seconds maximum) is the primary measure of interest.

### The FHS Offspring DNA Methylation Sample

The FHS tracks the development of cardiovascular disease in 3 generations of families recruited in Framingham, Massachusetts, beginning in 1948.^35^ We analyzed data from the second generation of study participants, who were recruited beginning in 1971. They are known as the Offspring Cohort.^36^ To be included in the DNA methylation study, participants must have attended the Framingham Offspring 8th follow-up visit during 2005 and 2008 and have provided a buffy coat sample.

### DNA Methylation Data

Data were normalized using the “dasen” method in the *wateRmelon R* package and subjected to downstream QC. Samples with missing rate >1% at *p* < 0.01, poor SNP matching to the 65 SNP control probe locations, and outliers by multidimensional scaling techniques were excluded. Probes with missing rate of >20% at *p* < 0.01 were also excluded. Additional information on DNA methylation, normalization, and quality control is available in the work of Mendelson et al.^37^

A flowchart documenting the number of samples at each stage of data preparation is shown in eFigure 1B (links.lww.com/WNL/C174).

### Dementia Diagnosis

As previously published,^38-40^ participants in this cohort have been assessed at each examination with the MMSE and flagged for further examinations if (1) they were identified as having possible cognitive impairment on the basis of screening assessments, (2) when subjective cognitive decline was reported by the participant or a family member, (3) on referral by a treating physician or by ancillary investigators of the FHS, or (4) after review of outside medical records. All cases of possible cognitive decline and dementia were reviewed to determine the presence of dementia, as well as dementia subtype and date of onset.^39^

Dementia ascertainment in our data set extended through 2018 (dbGaP accession pht010750.v1.p13, data set vr_demsurv_2018_a_1281s). Dementia status was determined for 2,468 participants. Of this group, *N* = 2,264 were alive and free of dementia at DNA methylation baseline. This analysis sample contributed a maximum of 14 years of follow-up time for dementia ascertainment, over which interval n = 151 (64 men and 87 women) developed dementia at an average age of 82 years (SD = 6).

### DNA Methylation Clock Estimation

In both ADNI and the FHS Offspring Cohort, we calculated 4 of the DNA methylation age (DNAmAge) clocks (Horvath, Hannum, PhenoAge, and GrimAge) using the online calculator found at dnamage.genetics.ucla.edu/new. The “normalization” and “advanced analysis in blood” options were selected, and data were anonymized before upload. From the results file, we extracted the corresponding DNA methylation age calculations (DNAmAge, DNAmAgeHannum, DNAmPhenoAge, and DNAmGrimAge) along with the estimates of white blood cell type abundance. DunedinPACE was calculated in *R* following the procedures described in the work of Belsky et al.^17^ To account for potential technical confounding introduced during DNA methylation measurement (e.g., differential reaction efficiency between batches of assays), values of the 5 algorithms were residualized for the DNA plate number. Finally, to derive estimates of DNA methylation age advancement, these values were further residualized for chronologic age at the time of the DNA assessment.

### Statistical Analysis

All analyses were conducted in *R*, except for Cox proportional regression analyses in the FHS Offspring Cohort which were conducted in STATA. All regression models were adjusted for sex. To enable effect size comparisons, all age-residualized scores were standardized to mean = 0, SD = 1 before analysis. In ADNI, we calculated Huber-White robust standard errors using the *plm* and *lmtest* packages in *R* to account for the fact that some individuals contribute more than 1 time point as described in the ADNI DNA Methylation Sample. In FHS, effect sizes are reported as hazard ratios (HRs) per SD increment of the aging measures estimated from Cox proportional hazard regression. To adjust means for sex, we calculated least-squares means with proportional weights in the *lsmeans* package in *R.* To account for technical variation, we also tested models adjusted for white blood cell abundance (plasmablasts, +CD8pCD28nCD45RA-T cells, naïve CD8 T cells, CD4 T cells, natural killer cells, monocytes, and granulocytes^13,41^). All analyses were performed in parallel by a second, independent researcher to confirm reproducibility.

### Standard Protocol Approvals, Registrations, and Patient Consents

All research activities were approved by Institutional Review Boards at the participating study sites. Participants provided written informed consent.

### Data Availability

All data used in this report are publicly available; access is granted after application approval from the relevant study's research review committee (ADNI: adni.loni.usc.edu/data-samples/access-data/; FHS Offspring Cohort: ncbi.nlm.nih.gov/projects/gap/cgi-bin/study.cgi?study_id=phs000724.v9.p13).

## Results

### DNA Methylation Measures of Aging in ADNI

DNA methylation data were available for 649 individuals and 1,706 samples (mean [SD] age at first DNA collection = 74.77 (7.66), male = 55.6%). The mean education of the 649 individuals was 16.22 years (SD = 2.71), and the majority self-identified as White (*N* = 636, 98.0%). Table 1 describes characteristics of participants in ADNI.

**Table 1:**
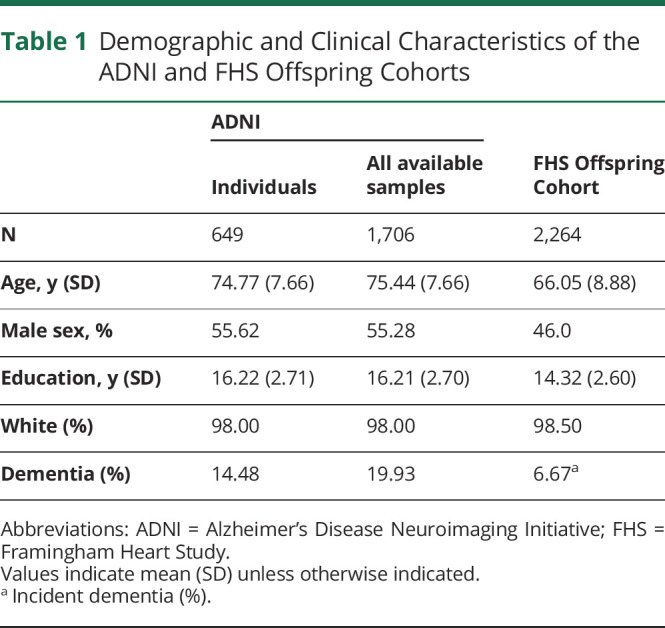
Demographic and Clinical Characteristics of the ADNI and FHS Offspring Cohorts

**Figure 1:**
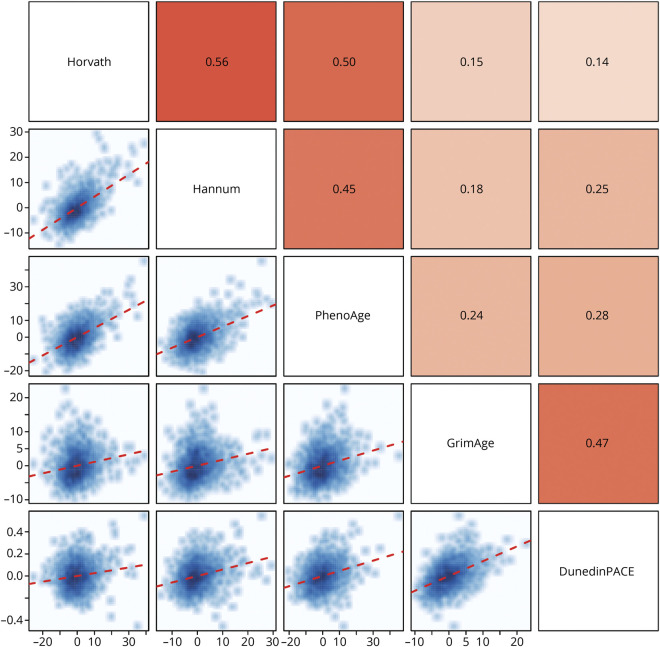
Correlations Between the 5 DNA Methylation Measures of Aging in ADNI The matrix above the diagonal plots the Pearson *r* statistic (with cell color depicting magnitude from light = low to dark = high), while the matrix below the diagonal shows the scatterplots for each comparison. The red dotted line describes the linear regression line. Correlations are adjusted for sex.

Table 2 contains descriptive data (mean [SD] and range) about the 5 measures of DNA methylation aging. The Horvath, Hannum, PhenoAge, and GrimAge clocks are measured in units of chronologic years, and DunedinPACE is measured in years of physiologic decline per 1 chronologic year. All DNA methylation measures of aging were associated with sex; males had older DNA methylation age on the clocks and faster DunedinPACE. All the following analyses include sex as a covariate. Similarly, all DNA methylation measures of aging were correlated with chronologic age such that chronologically older individuals appeared to have older DNA methylation age on the clocks and faster DunedinPACE (ranging from *r* = 0.30 for DunedinPACE to *r* = 0.85 for GrimAge). Going forward, we use measures of DNA methylation age *advancement*, derived by residualizing the measures described in Table 2 for participant age at the time of DNA data collection, rendering them uncorrelated with age. Figure 1 shows the correlations between the measures of DNA methylation age advancement. The measures were significantly intercorrelated; the largest correlations were observed between the first-generation clocks and PhenoAge (*r =* 0.45–0.56) and between GrimAge and DunedinPACE (*r* = 0.47); otherwise, correlations ranged from *r =* 0.14 to *r =* 0.28.

**Table 2:**
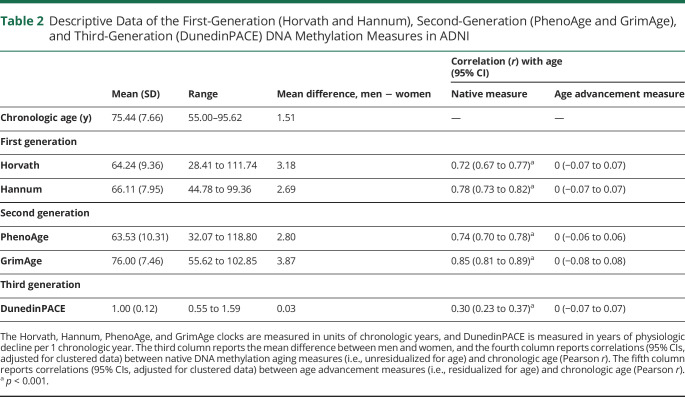
Descriptive Data of the First-Generation (Horvath and Hannum), Second-Generation (PhenoAge and GrimAge), and Third-Generation (DunedinPACE) DNA Methylation Measures in ADNI

### Association Between DNA Methylation Measures of Aging and Dementia Diagnosis in ADNI

**Figure 2:**
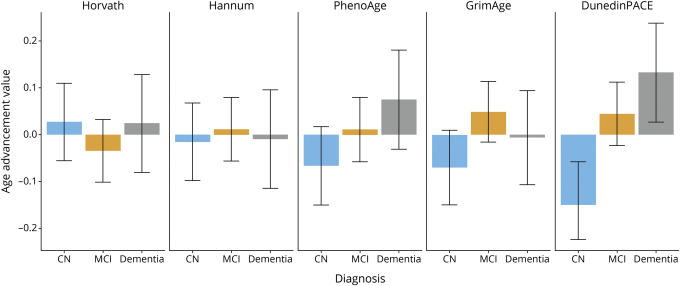
Mean DNA Methylation Age Advancement Values in Aging in ADNI Within Each of the 3 Diagnostic Categories Values are grouped by diagnostic category at the time of interview: CN (blue bars), MCI (gold bars), and Dementia (grey bars). The 3 diagnostic status groups did not differ significantly from one another on either of the first-generation DNA methylation clocks (Horvath and Hannum clocks) or on the second-generation clocks (PhenoAge and GrimAge). By contrast, individuals with MCI or Dementia had faster DunedinPACE scores than those who were CN. Bars represent means, and whiskers represent 95% CIs. Values are standardized to mean = 0, SD = 1. CN = cognitively normal; MCI = mild cognitive impairment.

At each DNA data collection point, ADNI participants were categorized into 3 diagnostic groups. Figure 2 shows the mean values of the 5 DNA methylation measures of aging for the 3 diagnostic groups: CN, MCI, and Dementia (for comparison purposes, DNA methylation age advancement values have been standardized to mean = 0 and SD = 1). The 3 diagnostic groups did not differ significantly from one another on first-generation clocks (Horvath and Hannum) or second-generation clocks (PhenoAge and GrimAge). By contrast, we observed an ordered association between diagnoses of CN, MCI, and Dementia for DunedinPACE: Individuals with a diagnosis of MCI (beta = 0.19, 95% CI: 0.03–0.34) and, to a greater extent, individuals with a diagnosis of Dementia (beta = 0.28, 95% CI: 0.08–0.47) had significantly faster DunedinPACE scores than CN individuals (see eTable 1, links.lww.com/WNL/C174, for details).

### Association Between DNA Methylation Measures of Aging and Cognitive Function in ADNI

At each DNA collection, ADNI participants were given 3 cognitive screening tests: the ADAS-Cog-13, the MMSE, and the MoCA. Table 3, Panel A presents the associations between the 5 DNA methylation measures of aging and scores on these 3 cognitive screening tests. Neither of the first-generation DNA methylation clocks nor GrimAge was associated with scores on the ADAS-Cog-13, MMSE, or MoCA (beta = −0.03 to 0.03). By contrast, advanced PhenoAge and faster DunedinPACE scores were both associated with worse scores on ADAS-Cog-13 (beta = 0.07 to 0.08) as well as MMSE and MoCA (beta = −0.06 to −0.10), indicating greater cognitive impairment.

**Table 3:**
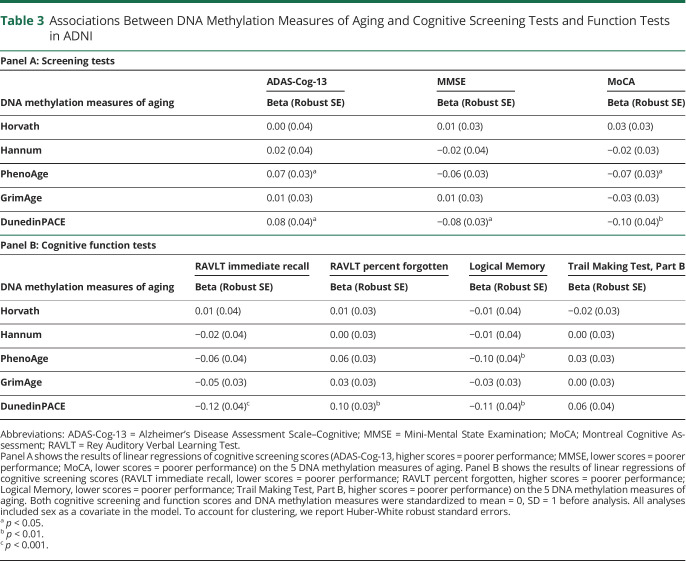
Associations Between DNA Methylation Measures of Aging and Cognitive Screening Tests and Function Tests in ADNI

**Figure 3:**
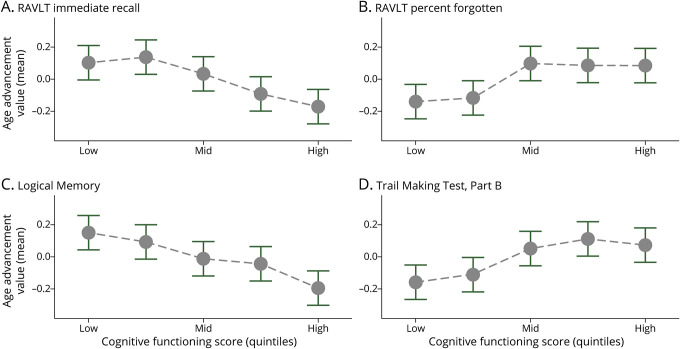
DunedinPACE Values by Test Score Quintile for the Rey Auditory Verbal Learning Test, Logical Memory Test, and Trail Making Test Cognitive Assessments in Aging in ADNI Faster DunedinPACE was associated with poorer learning and memory (RAVLT, immediate recall [A] and percent forgotten [B]), episodic memory (Logical Memory test [C]), and executive functioning (Trail Making Test, Part B [D]). Cognitive function scores (x-axis) are binned into quintiles (1–5); grey dots represent mean age advancement value, and whiskers represent 95% CIs. The y-axis represents DunedinPACE (age-residualized, adjusted for sex, and standardized to mean = 0, SD = 1).

ADNI participants were also administered a battery of cognitive function tests. Table 3, Panel B presents the associations between the 5 DNA methylation measures of aging and 4 measures of cognitive functioning: Rey Auditory Verbal Learning Test (both learning and memory), Logical Memory test (episodic memory), and Trail Making Test (executive function). Neither the first-generation clocks (Horvath and Hannum) nor GrimAge was consistently associated with performance on these tests (beta = −0.05 to 0.01). By contrast, advanced PhenoAge and, to a greater extent, faster DunedinPACE scores were both associated with significantly worse learning (beta = −0.06 to −0.12), more forgetting (beta = 0.06 to 0.10), and worse episodic memory (beta = −0.10 to −0.11) (Figure 3 and eFigure 2, links.lww.com/WNL/C174).

### Sensitivity and Secondary Analyses

Associations reported here were robust in several sensitivity analyses (eTables 1–3, links.lww.com/WNL/C174). First, associations were robust to distributional assumptions. Both the dementia-screening tests and the cognitive function measures had distributions that deviated from normal. As such, we repeated all analyses comparing the results with the “native” (i.e., original) scores, log-transformed scores, and scores binned into quintiles. Regardless of how we handled the distributions, the results were comparable (eTables 2 and 3). Second, after controlling for abundance estimates of different types of white blood cells, associations between DunedinPACE and clinical diagnoses and cognitive function tests were smaller but remained statistically significant at the alpha = 0.05 level (eTables 1 and 3), whereas those with the dementia-screening tests fell short of significance (eTable 2). It is important that Pace of Aging, on which DunedinPACE was trained in an independent sample,^16,17^ includes a longitudinal change in observed white blood cell abundance, making this an overcontrol. Finally, *APOE e4* is known to be associated with dementia risk; however, it was not associated with first-generation, second-generation, or third-generation DNA methylation measures of aging (eTable 4).

### Association Between DNA Methylation Measures of Aging and Dementia in the FHS Offspring Cohort: Replication and Extension

To replicate and extend the test of the association between DunedinPACE and dementia, we turned to the FHS Offspring Cohort. This longitudinal analysis included *N* = 2,264 participants with a maximum of 14 years of follow-up time for dementia ascertainment. Over this time interval, n = 151 (64 men and 87 women) developed dementia at an average age of 82 years (SD = 6).

Participants measured to have more advanced aging on the clocks and faster DunedinPACE at baseline were at increased risk of developing dementia over follow-up; the largest effect was for DunedinPACE (hazard ratio [HR] [95% CI] = 1.39 [1.21–1.61]), followed by PhenoAge, GrimAge, and Horvath (Table 4). As with ADNI, sensitivity analyses controlling for white blood cell abundance estimates attenuated effect sizes; only DunedinPACE (HR [95% CI] = 1.27 [1.07–1.49]) and the Horvath clock (HR [95% CI] = 1.21 [1.08–1.36]) significantly predicted risk of dementia at *p* < 0.05 (Table 4 and eFigure 3, links.lww.com/WNL/C174).

**Table 4:**
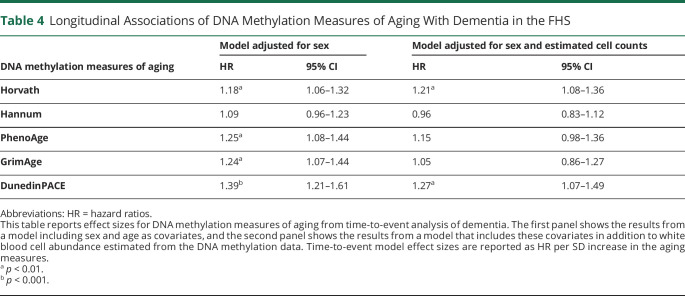
Longitudinal Associations of DNA Methylation Measures of Aging With Dementia in the FHS

## Discussion

Aging increases risk for Alzheimer disease, related dementias, and cognitive impairment.^42^ Moreover, the majority of cases occur later in life and for such individuals, unlike those with the less common familial AD, aging represents the largest risk factor for dementia.^43^ The potential to capture the individual dynamics that define the risk of cognitive decline attributable to *biological aging* is of great interest to gerontologists and clinicians alike. In this report, using data from ADNI and the FHS Offspring Cohort, we compared associations between first-generation, second-generation, and third-generation DNA methylation measures of aging and multiple measures of cognitive aging and dementia. When evaluated against clinical screening test scores, measures of cognitive functioning, and a clinical diagnosis of dementia, the third-generation DunedinPACE measure was more predictive than earlier generations of clocks. In ADNI, it was the only biological aging estimate to show consistent associations with every measure of cognitive impairment tested in the predicted direction of faster aging and more impairment. Moreover, faster DunedinPACE was associated with increased risk of developing future dementia in the FHS Offspring Cohort.

A DNA methylation algorithm that can assess biological aging should be robustly associated with cognitive dysfunction characteristic of AD/ADRD. First, we showed that individuals with a diagnosis of dementia and, to a lesser extent, mild cognitive impairment had faster DunedinPACE compared with individuals who were CN. This pattern was not observed for the first-generation and second-generation DNA methylation age advancement clocks. Second, individuals who scored poorly on screening measures commonly used in memory clinics (ADAS-Cog-13, MMSE, and MoCA) had older DNA methylation age advancement (assessed by PhenoAge) and faster DunedinPACE. Third, individuals' worse cognitive function was associated with older DNA methylation age advancement (assessed by PhenoAge) and faster DunedinPACE. It is important to note that the cognitive measures that we examined overlap to some extent, for example, the Logical Memory test is used to derive AD diagnoses. However, we think it essential to present evidence from all the cognitive measures because different studies often evaluate different cognitive measures, making it difficult to compare studies and reconcile inconsistencies.

Previous studies testing associations between DNA methylation clocks and late-life cognition and dementia have yielded equivocal and inconsistent evidence.^21,44-46^ By contrast, this study suggests that the newer generation DunedinPACE measure is consistently associated with multiple manifestations of age-related cognitive deficits. This is consistent with previously reported evidence that faster DunedinPACE is associated with greater cognitive decline during midlife.^17^ This consistency suggests that vulnerability to cognitive impairment that is the hallmark of risk for dementia can be captured by considering how fast a person is aging biologically compared with their age peers. The finding that extremely fast DunedinPACE scores occur with dementia is consistent with the view that dementia is not part of normal aging.

Consistent with previous studies (e.g., references 17-19,24,47,48), the 5 tested DNA methylation measures of aging vary in the extent to which they are intercorrelated, and clocks in the same generation tend to be more highly correlated with one another. This suggests that although different DNA methylation measures of aging capture some common elements, they are also clearly distinct. First-generation clocks were trained to predict chronologic age. This approach is based on the assumption that differences in DNA methylation between older and younger people represent biological processes of aging. Second-generation clocks were trained to predict mortality, using physiologic variables as intermediates. This approach is based on the assumption that differences in DNA methylation between people with higher as compared with lower risk for mortality represent biological processes of aging. The third-generation DunedinPACE was trained to predict biological change between ages 26 and 45 years in a same-age cohort. This approach is based on the assumption that DNA methylation differences between people experiencing slower as compared with more rapid decline in the function of multiple organ systems represent biological processes of aging. The evidence presented here suggests that progressive generations of clocks may be more sensitive predictors of cognitive outcomes. Moreover, the association of DunedinPACE with dementia recommends midlife prevention if some patients' course toward dementia begins in midlife.

There are caveats and limitations. First, despite robust associations between DunedinPACE and measures of cognitive aging, none of the currently available measures of DNA methylation aging match clinically validated risk markers of ADRD on strength-of-association. For example, within the ADNI participants analyzed in the present report, individuals with a diagnosis of dementia were 12 times more likely to carry 2 *APOE ε4* alleles than individuals who were CN, an effect size of a 0.94 SD-unit difference between dementia vs CN. To put the effect size for the DunedinPACE comparison between dementia vs CN in perspective, it yielded a 0.28 SD-unit difference. Second, most of the participants are White because of the lack of ethnic diversity of the participants enrolled in ADNI and Framingham. Initial evidence shows that an earlier version of a methylation Pace of Aging algorithm, DunedinPoAm, is associated with poorer physical health among both Black and White participants,^19^ but more research is needed on this front. Third, we were able to report only cross-sectional associations between DNA methylation measures of aging and cognitive impairment and AD in ADNI because the number transitioning to a new diagnosis was too small for statistical power among ADNI participants who had methylation data. To overcome this limitation, we extended our analysis to the larger FHS Offspring Cohort and found that DunedinPACE was associated prospectively with future risk of developing dementia. Fourth, this study reports initial replicated evidence that DunedinPACE derived in midlife signals dementia risk in late life, but life-course longitudinal studies should evaluate potential causal pathways including early-life age accelerators (e.g., low socioeconomic status, low education, and smoking) and potential late-life mediators (e.g., disease multimorbidity^17^). Fifth, dementia is also not a single disease and future, adequately powered studies with dementia subtypes should test for DunedinPACE's specificity. Sixth, ample evidence points to genetic loci contributing to dementias.^49^ By contrast, DNA methylation variation represents the epigenetic results of processes along pathways toward dementia, suggesting DunedinPACE is best considered a noncausal risk indicator.

As the search gains steam for geroscience-guided interventions that might slow aging and prevent the onset of age-related diseases, including Alzheimer's disease and related dementias, the need for reliable measures of biological aging related to dementia is becoming more apparent. Such measures could serve to identify people at high risk for future dementia and could serve as surrogate measures to evaluate interventions while waiting for the longer term outcome of dementia. DNA methylation measures of aging have offered promise, but their relation to cognitive aging and dementia has been equivocal. Here, we find evidence that a third-generation DNA methylation measure of aging, trained on longitudinally measured biological decline, may prove useful in dementia research.

## Glossary

AD: Alzheimer disease
ADAS-Cog-13: Alzheimer's Disease Assessment Scale–Cognitive
ADNI: Alzheimer's Disease Neuroimaging Initiative
ADRD: Alzheimer Disease and Related Dementias
CN: cognitively normal
FHS: Framingham Heart Study
HR: hazard ratio
MCI: mild cognitive impairment
MMSE: Mini-Mental State Examination
MoCA: Montreal Cognitive Assessment
SNP: Single Nucleotide Polymorphism
